# Esophageal Injury in Patients with Ankylosing Spondylitis After Cervical Spine Trauma: Our Case Series and Narrative Review

**DOI:** 10.3390/medicina61101855

**Published:** 2025-10-16

**Authors:** Nenad Koruga, Alen Rončević, Mario Špoljarić, Tomislav Ištvanić, Stjepan Ištvanić, Vedran Farkaš, Klemen Grabljevec, Anđela Grgić, Tatjana Rotim, Tajana Turk, Domagoj Kretić, Anamarija Soldo Koruga

**Affiliations:** 1 Department of Neurosurgery, University Hospital Center Osijek, 31000 Osijek, Croatia; 2 Faculty of Medicine, Josip Juraj Strossmayer University of Osijek, 31000 Osijek, Croatia; 3 Department of Vascular Surgery, University Hospital Center Osijek, 31000 Osijek, Croatia; 4 Department for Rehabilitation of Patients After Traumatic Brain Injury, University Rehabilitation Institute, 1000 Ljubljana, Slovenia; 5 Department of Physical Medicine and Rehabilitation, Clinical Hospital Centre Osijek, 31000 Osijek, Croatia; 6 Department of Anatomy and Neuroscience, Faculty of Medicine, University of Osijek, 31000 Osijek, Croatia; 7 Department of Diagnostic and Interventional Radiology, University Hospital Center Osijek, 31000 Osijek, Croatia; 8 Department of Neurology, University Hospital Center Osijek, 31000 Osijek, Croatia

**Keywords:** ankylosing spondylitis, esophageal injuries, spinal injuries, spinal fusion, fluoroscopy

## Abstract

*Introduction*: Ankylosing spondylitis (AS) is a chronic inflammatory disorder that causes progressive ossification and fusion of the spine, particularly in the cervical region. This results in a rigid spinal column that is highly susceptible to unstable fractures, even after low-energy trauma. Cervical fractures in AS are often complex, extending through multiple spinal segments, and are associated with a high risk of neurological compromise. Esophageal injury associated with such fractures is rare but clinically significant, as the anatomical vicinity of the esophagus makes it vulnerable to direct trauma, delayed perforation, or secondary damage from fracture displacement and hardware failure. *Aim*: The purpose of this review is to present and highlight the clinical relevance of esophageal injury in cervical spine trauma among patients with AS, emphasizing the diagnostic challenges and surgical treatment in order to improve outcomes. *Results*: Esophageal injuries in the context of AS-related cervical trauma are frequently overlooked due to subtle clinical manifestations such as dysphagia, subcutaneous emphysema, or covert signs of mediastinitis. Plain radiographs are insufficient to identify such complications; advanced imaging modalities are often required for detection. Management is complex and usually demands a multidisciplinary approach, involving both stabilization of the cervical spine and repair of the esophagus. Despite treatment efforts, these patients remain at increased risk for morbidity and mortality, mainly due to infection and sepsis. *Conclusions*: Esophageal injury in cervical spine trauma associated with AS is an uncommon but life-threatening condition. Early recognition, comprehensive radiologic evaluation, and careful surgical planning are crucial for optimal management. Heightened clinical suspicion and awareness of this rare complication are essential to improve diagnostic accuracy and patient outcomes.

## 1. Introduction

Ankylosing spondylitis (AS) is a seronegative arthropathy that results in pathological ossification of the ligaments, discs, endplates and apophyseal structures. Cervical spinal fractures are more common in patients with ankylosing spondylitis than in patients without ankylosing spondylitis due to coexistent osteoporosis and kyphotic alignment of the spine. The risk of fracture dislocation and associated spinal cord injury is significantly higher in these patients [1].

The ankylosed spine is not very resistant to trauma and the lesions affect the points of least resistance electively, essentially the syndesmophytic disc bridges at the level of the hinge zones of the spine [1]. As an autoimmune disease, AS mainly affects axial bones, resulting in rigidity of the spine, especially lower cervical vertebrae, and often leads to unstable fractures of the cervical spine, mostly due to the spondylotic changes in all three columns of the cervical spine. Among AS patients, the lower cervical spine or cervico-thoracic junction are the most affected segments. Fatal injuries occur in about 30% of all patients and the estimated incidence of cervical spine injuries is up to 15% among AS patients. Prompt and adequate surgical treatment is essential to obtain motor function in patients and restore cervical spine alignment [2]. The management of dislocated fractures of the cervical spine in ankylosing spondylitis (AS) is particularly difficult and challenging. These lesions frequently involve the anterior and posterior elements of the spine, generating major instability and highly possibly lead to neurologic deficit. Surgical management is difficult because of the rigidity of the spine and the frequently associated osteoporosis. Different management strategies have been proposed, including anterior and posterior approaches and combined routes, with or without various external immobilizations [3]. The goal of this study was to show the benefits of surgical treatment regardless of surgical routes in the treatment of these fractures.

Diagnostics and Treatment: Patients suffering from AS with cervical spine trauma should be diagnostically assessed upon admission. The clinical decision-making process followed to achieve the right treatment depends on assessment protocols. Patients with AS who have sustained cervical trauma are at high risk for unstable fractures even with minimal trauma, due to ankylosed spines. When these patients are admitted to the hospital, the usual approach consists of initial assessment, which includes the ABCDE protocol; cervical spine immobilization, with a rigid collar or spine board; and neurologic assessment, including the Glasgow Coma Scale (GCS), American Spinal Injury Association (ASIA), and Frankel score. This is followed by radiologic imaging; CT scanning is the gold standard for detecting fractures of the entire spine, and MRI detects possible spinal cord compression or ligamentous, disc, or soft tissue injuries [4]. Further diagnosis includes the identification of fracture location, its stability, and the neurological status of the patient. Neurological deficit and instability are clear indicators for surgery; also, progressive deformity and conservative treatment failure are surgical indications. In an intact patient with stable fractures, conservative treatment, including bracing and medication, could be considered, but with close observation of the patient in terms of delayed instability.

Surgery: Recently, Tian et al. in 2023 [5] gave a complete overview regarding surgical treatment with proposed decision-making surgical approaches. According to their experience on over one hundred patients with AS and concomitant cervical trauma, they developed a scheme based on several trauma types: type 1—single-level fracture dislocation as the most frequent one; type 2—spinal cord injury without bony dislocation; and type 3—pseudoarthrosis, Andersson lesion [5]. Based on their classification, the surgical approach is a method of choice, especially in patients with cervical instability, neurological deficit, progressive deformity, or failure of conservative treatment; an anterior, posterior, or combined approach is commonly used. Post-surgical treatment and neurological surveillance and monitoring continue at the ICU. Once the patient is transferred to the ward, early rehabilitation is necessary to achieve better post-surgical recovery. Most of the encountered post-surgical complications are CSF leak, hardware failure, infection of the surgical site, or further neurological deterioration. Authors have encountered post-surgical complication of the hardware loosening and displacement with further injury of the esophagus, which requires re-surgery, i.e., the placement of a new anterior titanium plate and suturing of the esophagus.

## 2. Review of the Literature

**Figure 1:**
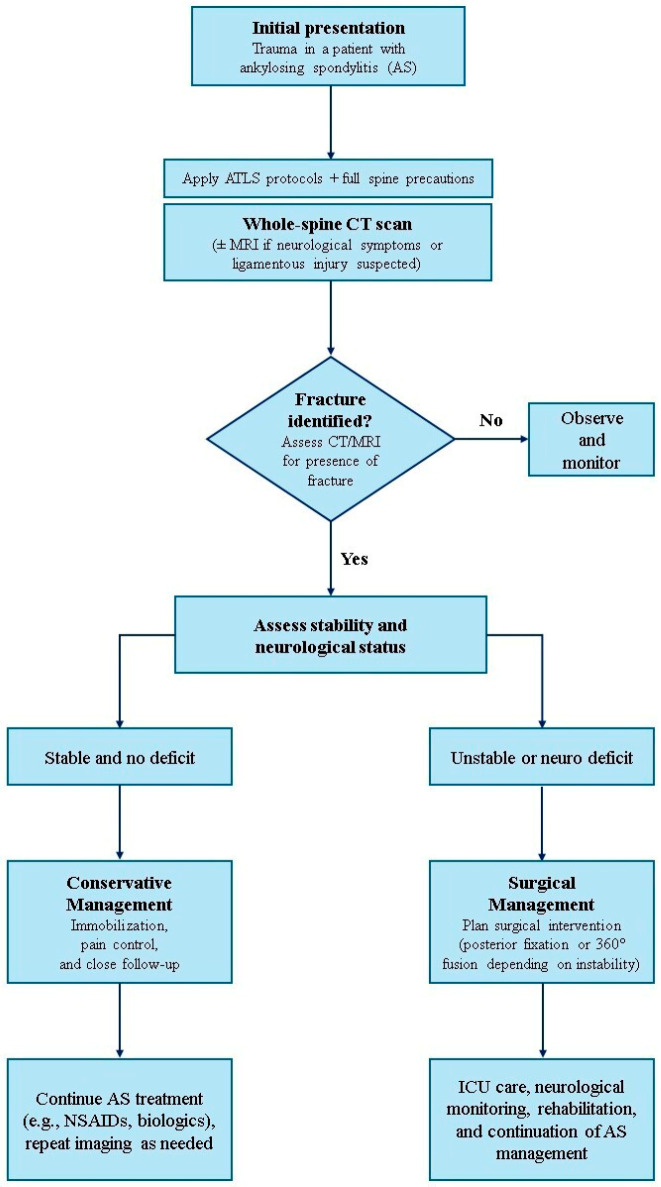
Point-by-point algorithm of clinical and radiologic evaluation and treatment of patients with ankylosing spondylitis after cervical spine trauma.

The authors conducted research in a timespan of ten years (from February 2016 to October 2025) using the Pubmed and Google scholar search engines and the following medical keywords: “ankylosing spondylitis” and “cervical spine trauma”. Several milestone papers emerged in which authors recognized and elucidated novel diagnostic and classification models and the possibilities of surgical approaches. In patients with AS, additional trauma of the neck causes further complications and dictates an individual treatment approach for each patient, as shown by Xiang et al., who conducted a nomogram prediction study that directs surgeons toward an adequate approach. According to these authors, a fracture was classified into three types based on the fracture line and severity: type I, disc injury; type II, vertebral body injury; and type III, vertebral body and disc injury. Four subtypes were also defined as follows: (A) fracture without dislocation, (B) fractures with dislocation without obvious bone defects, (C) fractures with obvious dislocation or severe bone gaps, and (D) fractures with epidural hematoma or CSF leakage [6]. According to these authors, several key factors have been highlighted; except surgical approaches and nomograms, a prompt radiologic evaluation—with a CT scan as a primary option and MRI for further radiologic evaluation—is necessary before the final treatment decision (Figure 1). Also, coexistence of the ossification of the posterior longitudinal ligament (OPLL) significantly increases complications and surgical risks [6].

Regarding complications, two studies, one prospective and the other a meta-analysis, revealed contrary results: A modest complication rate with 8% wound infection versus a complication rate of almost 20% [5,7].

**Figure 2:**
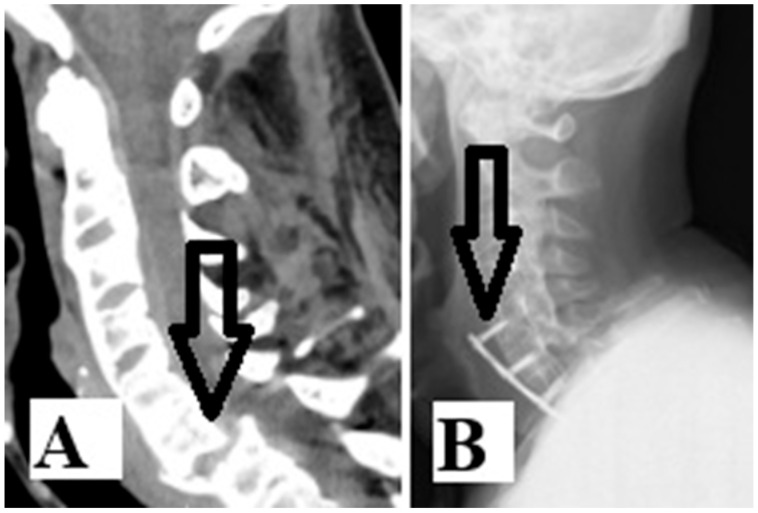
Sagittal CT scan (**A**) revealed a luxation fracture at the C6–C7 level (arrow). Follow-up X-rays scan after six months (**B**) revealed hardware failure (arrow), which led to the esophagus injury.

**Figure 3:**
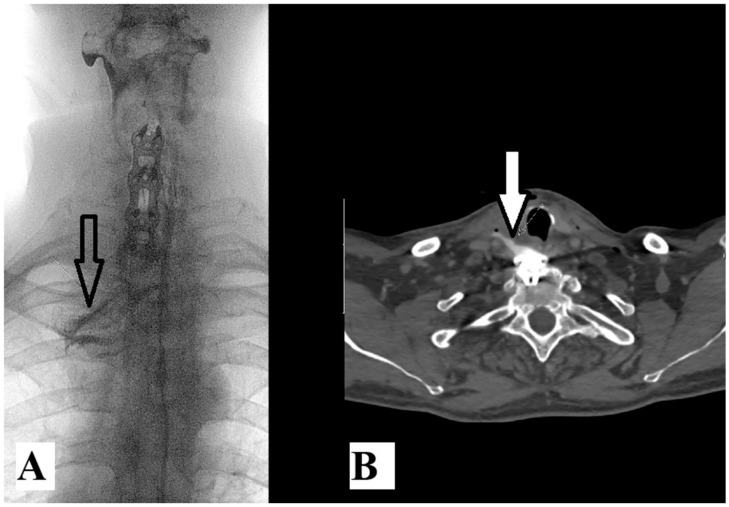
Fluoroscopy image of contrast media swallow test (**A**) reveals extralumination (arrow) of water-soluble contrast media through the perforated upper esophagus. Axial CT image (**B**) at the level of the first thoracic vertebral body showing extralumination (arrow) of previously swallowed water-soluble contrast media into the prevertebral and carotid space of the neck.

From another aspect, according to the same search engines and with the addition of the words “esophageal injury”, only three cases of AS with esophageal injury [8,9,10] emerged in the last ten years (Table 1). Injury of the esophagus is a rare but serious complication when cervical spine fracture occurs in patients with AS. There are two possible mechanisms of esophageal injury: direct or acute injury or delayed injury after hardware failure. Our patient had luxation fracture at the C6–C7 level (Figure 2A). He first underwent Crutchfield traction and anterior C5-T1 fixation with a titanium plate. Six months after surgery, the patient underwent re-surgery due to hardware failure and esophageal injury (Figure 2B). Further radiologic examination should be performed to confirm these devastating injuries (Figure 2B and Figure 3A).

We took into account cases in the last ten years. Nevertheless, the article from Zdichavsky et al., published in 2004 [11], was the most similar to our case in which authors described the failure of anteriorly placed hardware, i.e., its displacement ten months after surgery and concomitant injury of the esophagus in a 50-year-old male patient at the C6 C7 level. Also, two articles were published in 2022 and 2024; the first one, by Molina et al., was a retrospective review in which pharyngo-esophageal injury caused by the hardware extrusion was described in a timespan of seven and a half years after surgeries in 9 out of 4122 patients [12]. Male predominance (67%) was seen in their series, and all cervical levels were included; no specific level was displayed. In 2024, Hong et al. [13] presented an article in which 17 cases of AS with cervical fracture were described. Among their series, 16 out of 17 patients were men, and they performed fixation of the cervical spine exclusively using the anterior approach [2]. Moreover, they did not encounter any post-surgical complications. The authors’ research of the literature was limited to studies published in the period from 2016 to 2025 to capture the most recent advances in diagnostics, surgical techniques, and perioperative management of patients with AS and injury of the esophagus. This timeframe was selected to reflect contemporary clinical practice and reduce methodological variability associated with older studies.

## 3. Discussion

Ankylosing spondylitis is a chronic systemic inflammatory disorder that primarily targets the axial skeleton; it presents specific surgical challenges in these patients. This condition leads to ossification and fusion of the spinal segments, profoundly altering the biomechanics of the spine [14]. These changes, while initially driven by inflammatory processes, result in a spine that becomes increasingly rigid and highly susceptible to injury. Among the various regions of the spine affected by AS, the cervical spine is under particularly high risks when trauma occurs [13]. Moreover, in the context of trauma, the cervical spine in AS patients can be considered both pathologically fragile and functionally compromised. This disease progression results in the fusion of vertebral segments, creating a so-called “bamboo spine” due to the characteristic radiographic appearance [15]—this fusion results in mechanical disadvantage, especially to external forces. Even minor trauma, such as a ground-level fall, can translate into significant stress at focal points in the cervical spine, often leading to fractures [16]. This risk is compounded by the presence of osteoporosis, which is common in AS patients due to chronic inflammation, limited mobility, and altered bone remodelling [17]. Cervical spine injuries in AS are frequently underestimated or misdiagnosed due to subtle clinical presentations; limitations in conventional imaging, which in many patients is present with non-specific neck pain; and the absence or delay of neurological symptoms [18]. These cases are particularly dangerous, as unstable fractures may progress subtly and eventually lead to spinal cord injury [19]. Diagnosis of cervical spine injuries in AS patients is complicated due to natural and structural changes in the cervical spine induced by the disease. Despite being the first-line imaging modality in trauma, plain radiographs are highly unreliable [20], especially due to the altered anatomy, i.e., kyphotic deformities and ossified ligaments, which obscure fracture lines. Therefore, other imaging modalities, such as computed tomography (CT), have become necessary and essential in the evaluation of spinal trauma in AS [21]. Magnetic resonance imaging (MRI) as the other advanced technique is necessary in evaluating spinal cord involvement, ligamentous injury, or hematomas—mostly epidural [22]. The mechanism and pattern of injury of the cervical spine in AS patients significantly differs in comparison with the same injuries in patients with a normal spine; in AS patients, injuries extend throughout the entire vertebral segment and involve multiple columns [18]. Typical fractures in AS patients usually traverse the vertebral body and intervertebral discs in a horizontal fashion, which results in highly unstable injuries. These three-column fractures largely increase the risk of neurological compromise due to biomechanical instability and the possibility of its displacement [19]. A multidisciplinary approach is mandatory in the management of cervical spine trauma in patients with AS; initial stabilization is crucial but challenging [23]. It is necessary to avoid excessive manipulation and poorly executed transfers due to the injuries of a pre-existing deforming cervical spine, which can lead to further injuries to the cervical medulla and exacerbate the patient’s neurological status. Cervical collars at the initial evaluation of the patient do not offer definitive and adequate stabilization; therefore, early recognition of spinal injury and proper handling of the patient are essential [24]. The treatment of these patients starts with radiologic evaluation and conservative treatment: Crutchfield traction, bracing, and bed rest. If good alignment is seen on a follow-up CT scan after Crutchfield traction, the next step is surgery for definitive management. The goal is to restore spinal alignment, through the decompression of neural elements and adequate internal fixation of the spine. According to the biomechanical considerations of the spine, it is recommended to achieve long-segment fixation to achieve better stabilization, regardless of the approach, anterior, posterior, or combined. It is necessary to emphasize that there is no unique or “most proper” way for stabilization; we can say that it largely depends on the type of injury or cervical segment of the injury, as well as the surgeon’s definite evaluation [25]. Surgical management aims to obtain spine stability and is considered definitive treatment; nevertheless, conservative manners of treatments might lead to inherent instability and likely end up with non-union [18,26]. Besides spine stability and rigid fixation, surgical treatment aims to restore spinal alignment and decompress neural elements. A preferable surgical treatment option is long-segment fixation, but a combined approach is considered to provide maximal mechanical stability [16,27]. Injuries of the cervical spine in AS patients unquestionably lead to certain complications; neurological ones are unfortunately common—a narrow spinal canal in combination with potential displacement of unstable fractures and possible epidural hematomas predisposes patients to spinal cord injury [18,19]. Therefore, prompt surgical decompression is often required in cases with significant cord compression [22]. Besides neurological injuries, which need proper and prompt surgical approach, other complications might be generated by hardware failure and consequent complications such as injury of esophagus, mediastinitis, CSF leak, or infections [28,29,30]. Intraoperative complications mostly include cardiovascular issues, respiratory failure, or iatrogenic injury of the dura. Beyond immediate surgical concerns, rehabilitation also poses unique challenges due to prolonged recovery in these patients and its pre-existing functional limitations imposed by AS [31]. The goals of rehabilitation should be achieved early: early mobilization, respiratory therapy, and positioning. Later stages of early rehabilitation include further mobilization of the affected patient, prevention of complications, and psychosocial support. Later stages of rehabilitation include further follow-up, maintaining mental health, and possibly returning to daily activities [32,33].

From our experience, several clinical implications need to be addressed: First, the type of approach depends on the type of fracture, patient’s health and comorbidities, and surgeon expertise and experience. We can claim that a combined approach offers better stability; nevertheless, the surgical approach primarily depends on surgeon’s individual decisions. Surgical outcomes vary in terms of surgical approach and post-surgical complications. A posterior-only approach in patients with AS and cervical spine fracture is a viable treatment option with decreased time of surgery and decreased blood loss. A combined anterior and posterior approach yields better stability, but with a higher percentage of post-surgical complications. Cervical spine trauma in patients with ankylosing spondylitis represents a complex and high-risk clinical scenario; the structural rigidity and altered biomechanics of the ankylosed spine create predisposition for unstable fractures, even with minimal trauma [14]. Diagnosis could be delayed due to atypical presentations and limitations of conventional imaging [20]. Surgical management is mandatory to acquire proper stabilization of the spine. Besides surgical expertise, interdisciplinary coordination is necessary to achieve better outcomes in the treatment of these patients [27]. Authors must emphasize that the quality of the reviewed studies varied considerably; most publications were case reports, reflecting the rarity of cervical fractures with associated esophageal injury in AS. Due to the limited number of studies and their scarcity, the data found did not provide high-level evidence according to conventional hierarchies. Consequently, conclusions drawn from the available data should be interpreted with caution. According to the extremely low number of patients with AS and concomitant cervical spine injuries, followed by even less occasions with esophageal injuries, we can conclude that there is no uniform approach in the treatment of these patients. The diagnostics and surgical treatment of these patients should be tailored and optimized individually by the surgeon and multidisciplinary team.

Limitations: Our study has several limitations that should be acknowledged. First, the number of reported cases remains small due to the rarity of esophageal injury following cervical spine trauma in patients with AS. Therefore, our conclusions are based mostly on limited clinical experience and published case reports or small retrospective series rather than high-level evidence. Second, the literature review was narrative rather than systematic; although we applied a focused search strategy, we cannot exclude the possibility of missing relevant publications, particularly those not indexed in major databases or published in languages other than English. Third, the included studies were heterogeneous in their details, which precluded quantitative comparison or meta-analysis. Fourth, our analysis did not include a direct comparison with patients who sustained cervical spine trauma without AS, as such comparative studies are extremely scarce and differ markedly in biomechanics and treatment algorithms and are inapplicable for comparison. Finally, given the retrospective nature of both the literature data and our institutional experience, the assessment of postoperative outcomes and complications may be influenced by reporting bias. Despite all limitations, this review provides a concise synthesis of available evidence on a rare but clinically significant complication, highlighting diagnostic challenges, surgical considerations, and areas for future research.

## 4. Conclusions

Injuries of the esophagus following cervical trauma in patients with ankylosing spondylitis are exceptionally rare but can lead to serious clinical consequences and the high risk of associated visceral injuries. The usual subtle symptoms of esophageal injury in these patients are frequently missed, and delayed diagnosis requires meticulous radiologic imaging and multidisciplinary treatment; therefore, early recognition, prompt radiological evaluation, and coordinated multidisciplinary management are critical to reducing morbidity and mortality. By maintaining a high index of suspicion and adhering to best-practice guidelines, clinicians can improve outcomes for this vulnerable patient population and help mitigate the potentially devastating consequences of cervical spine trauma in patients with ankylosing spondylitis.

## Figures and Tables

**Table 1:** Cases of esophageal injury published in a timespan of ten years.

Year/Author	Patient/Level	Type of Injury
2016, Li et al. [8]	M 43, C 6–C 7	Delayed, 3 days post trauma
	M 77, C 7 fracture	Delayed, 2 days post trauma
2016, Chen et al. [9]	M 61, C 6 fracture	Acute
2018, Vonhoff et al. [10]	M 66, C 6 fracture	Acute
2025, our study	M 51, C 6–C 7	Delayed, six months after surgery

## References

[1] Reinhold M., Knop C., Kneitz C., Disch A. (2018). Spine Fractures in Ankylosing Diseases: Recommendations of the Spine Section of the German Society for Orthopaedics and Trauma (DGOU). Global Spine J..

[2] Chen W., Yang Y., Pan W., Lei X., Hong Z., Luo H. (2024). Treatment of lower cervical spine fracture with ankylosing spondylitis by simple long anterior cervical plate: A retrospective study of 17 cases. Front. Neurol..

[3] Peng C., Luan H., Liu K., Song X. (2024). Comparison of Posterior Approach and Combined Anterior-Posterior Approach in the Treatment of Ankylosing Spondylitis Combined With Cervical Spine Fracture: A Systematic Review and Meta-Analysis. Global Spine J..

[4] Shaharudin N.S., Dunseath O.A., Azmi N., Aun N.Y. (2025). The Initial Assessment and Management of Cervical Spine Injuries: A Comprehensive Review. Cureus.

[5] Liu B., Yang Z., Ji H., Zhou F., Li W., Zhang Z., Tian Y. (2023). A Novel Classification of Cervical Spine Trauma in Ankylosing Spondylitis and Corresponding Surgical Outcomes. Orthop. Surg..

[6] Shen N., Wu X., Guo Z., Yang S., Liu C., Guo Z., Yang S.Y., Xing D., Chen B., Xiang H. (2022). Classification and Treatment for Cervical Spine Fracture with Ankylosing Spondylitis: A Clinical Nomogram Prediction Study. Pain. Res. Manag..

[7] Alam M.S., Hasan M.Z., Choudhury A.A.M., Jahan M.S., Dastagir O., Amin Molla M.R., Islam M.A. (2024). A Comprehensive Analysis of Surgical Outcomes for Spinal Fractures in Patients With Ankylosing Spondylitis: A 13-Year Prospective Study. Cureus.

[8] Qian S.J., Ye X.S., Chen W.S., Li W.L. (2016). Missed diagnosis of oesophageal perforation in ankylosing spondylitis cervical fracture: Two case reports and literature review. J. Int. Med. Res..

[9] Wang J., Shi L., Chen H., Yuan W. (2016). Esophageal Perforation in a Cervical Fracture Patient With Progressed Ankylosing Spondylitis: Case Report and Review of the Literature. Spine.

[10] Vonhoff C.R., Scandrett K., Al-Khawaja D. (2018). Minor Trauma in Ankylosing Spondylitis Causing Combined Cervical Spine Fracture and Esophageal Injury. World Neurosurg..

[11] Zdichavsky M., Blauth M., Bosch U., Rosenthal H., Knop C., Bastian L. (2004). Late esophageal perforation complicating anterior cervical plate fixation in ankylosing spondylitis: A case report and review of the literature. Arch. Orthop. Trauma. Surg..

[12] Yahanda A.T., Pennicooke B., Ray W.Z., Hacker C.D., Kelly M.P., Dorward I.G., Santiago P., Molina C.A. (2022). Pharyngoesophageal Damage from Hardware Extrusion at an Average of 7.5 Years After Anterior Cervical Diskectomy and Fusion: A Case Series, Discussion of Risk Factors, and Guide for Management. World Neurosurg..

[13] El Tecle N.E., Abode-Iyamah K.O., Hitchon P.W. (2015). Management of spinal fractures in patients with ankylosing spondylitis. Clin. Neurol. Neurosurg..

[14] Braun J., Sieper J. (2007). Ankylosing spondylitis. Lancet.

[15] Jurik A.G. (2011). Imaging the spine in arthritis—A pictorial review. Insights Imaging.

[16] Rustagi T., Drazin D., Oner C., York J., Schroeder G.D., Vaccaro A.R., Oskouian R.J., Chapman J.R. (2017). Fractures in spinal ankylosing disorders: A narrative review of disease and injury types, treatment techniques, and outcomes. J. Orthop. Trauma..

[17] Avouac J., Koumakis E., Toth E., Meunier M., Maury E., Kahan A., Cormier C., Allanore Y. (2012). Increased risk of osteoporosis and fracture in women with systemic sclerosis: A comparative study with rheumatoid arthritis. Arthritis Care Res..

[18] Westerveld L.A., Verlaan J.J., Oner F.C. (2009). Spinal fractures in patients with ankylosing spinal disorders: A systematic re-view of the literature on treatment, neurological status and complications. Eur. Spine J..

[19] Caron T., Bransford R., Nguyen Q., Agel J., Chapman J., Bellabarba C. (2010). Spine fractures in patients with ankylosing spinal disorders. Spine.

[20] Anwar F., Al-Khayer A., Joseph G., Fraser M.H., Jigajinni M.V., Allan D.B. (2011). Delayed presentation and diagnosis of cervi-cal spine injuries in long-standing ankylosing spondylitis. Eur. Spine J..

[21] Kouyoumdjian P., Guerin P., Schaelderle C., Asencio G., Gille O. (2012). Fracture of the lower cervical spine in patients with ankylosing spondylitis: Retrospective study of 19 cases. Orthop. Traumatol. Surg. Res..

[22] Leone A., Marino M., Dell’Atti C., Zecchi V., Magarelli N., Colosimo C. (2016). Spinal fractures in patients with ankylosing spondylitis. Rheumatol. Int..

[23] Whang P.G., Goldberg G., Lawrence J.P., Hong J., Harrop J.S., Anderson D.G., Albert T.J., Vaccaro A.R. (2009). The management of spinal injuries in patients with ankylosing spondylitis or diffuse idiopathic skeletal hyperostosis: A comparison of treatment methods and clinical outcomes. J. Spinal Disord. Tech..

[24] Taggard D., Traynelis V.C. (2009). Neurological deterioration after manipulation in undiagnosed ankylosing spondylitis cervical fracture. Eur. Spine J..

[25] Donnarumma P., Bozzini V., Rizzi G., Berardi A., Merlicco G. (2017). Surgical management of C-type subaxial cervical fractures using cervical traction followed by anterior cervical discectomy and fusion within 12 h after the trauma. J. Craniovertebr. Junction Spine.

[26] Taggard D.A., Traynelis V.C. (2008). Management of cervical spinal fractures in ankylosing spondylitis with posterior fixation. Neurosurg. Focus..

[27] Sapkas G., Kateros K., Papadakis S.A., Galanakos S., Brilakis E., Machairas G., Katonis P. (2009). Surgical outcome after spinal fractures in patients with ankylosing spondylitis. BMC Musculoskelet. Disord..

[28] Robinson Y., Willander J., Olerud C. (2015). Surgical Stabilization Improves Survival of Spinal Fractures Related to Anky-losing Spondylitis. Spine.

[29] Olerud C., Frost A., Bring J. (1996). Spinal fractures in patients with ankylosing spondylitis. Eur. Spine J..

[30] Mizutani Y., Okuda A., Maegawa N., Tada Y., Takano K., Asai H., Watanabe T., Kawai Y., Shigematsu H., Urisono Y. (2021). Esophageal incarceration associated with cervical vertebral fracture in a patient with diffuse idiopathic skeletal hyperostosis. J. Orthop. Sci..

[31] Kobayashi K., Imagama S., Sato K., Kato F., Kanemura T., Yoshihara H., Sakai Y., Shinjo R., Hachiya Y., Osawa Y. (2018). Postoperative Complications Associated With Spine Surgery in Pa-tients Older Than 90 Years: A Multicenter Retrospective Study. Global Spine J..

[32] Ramiro S., Nikiphorou E., Sepriano A., Ortolan A., Webers C., Baraliakos X., Landewe R.B.M., Van den Bosch F.E., Boteva B., Bremander A. (2023). ASAS-EULAR recommendations for the management of axial spondyloarthritis: 2022 update. Ann. Rheum. Dis..

[33] Millner J.R., Barron J.S., Beinke K.M., Butterworth R.H., Chasle B.E., Dutton L.J., Lewington M.A., Lim E.G.S., Morley T.B., O’Reilly J.E. (2016). Exercise for ankylosing spondylitis: An evidence-based consensus statement. Semin. Arthritis Rheum..

